# 3D-Printable Gelatin Methacrylate-Xanthan Gum Hydrogel Bioink Enabling Human Induced Pluripotent Stem Cell Differentiation into Cardiomyocytes

**DOI:** 10.3390/jfb15100297

**Published:** 2024-10-05

**Authors:** Virginia Deidda, Isabel Ventisette, Marianna Langione, Lucrezia Giammarino, Josè Manuel Pioner, Caterina Credi, Federico Carpi

**Affiliations:** 1 Department of Industrial Engineering, University of Florence, 50139 Florence, Italy; virginia.deidda@unifi.it (V.D.); isabel.ventisette@unifi.it (I.V.); 2 Department of Experimental and Clinical Medicine, University of Florence, 50134 Florence, Italy; marianna.langione@unifi.it; 3 Department of Neurosciences, Psychology, Drug Research and Child Health, University of Florence, 50139 Florence, Italy; lucrezia.giammarino@unifi.it; 4 Department of Biology, University of Florence, 50019 Sesto Fiorentino, Italy; josemanuel.pioner@unifi.it; 5 European Laboratory for Non-Linear Spectroscopy, University of Florence, 50019 Sesto Fiorentino, Italy; 6 National Institute of Optics, National Research Council, 50019 Sesto Fiorentino, Italy

**Keywords:** 3D bioprinting, hydrogel, HiPSCs, gelatin, GelMA, xanthan gum, cardiac, tissue engineering

## Abstract

We describe the development of a bioink to bioprint human induced pluripotent stem cells (hiPSCs) for possible cardiac tissue engineering using a gelatin methacrylate (GelMA)-based hydrogel. While previous studies have shown that GelMA at a low concentration (5% *w*/*v*) allows for the growth of diverse cells, its 3D printability has been found to be limited by its low viscosity. To overcome that drawback, making the hydrogel both compatible with hiPSCs and 3D-printable, we developed an extrudable GelMA-based bioink by adding xanthan gum (XG). The GelMA-XG composite hydrogel had an elastic modulus (~9 kPa) comparable to that of cardiac tissue, and enabled 3D printing with high values of printing accuracy (83%) and printability (0.98). Tests with hiPSCs showed the hydrogel’s ability to promote their proliferation within both 2D and 3D cell cultures. The tests also showed that hiPSCs inside hemispheres of the hydrogel were able to differentiate into cardiomyocytes, capable of spontaneous contractions (average frequency of ~0.5 Hz and amplitude of ~2%). Furthermore, bioprinting tests proved the possibility of fabricating 3D constructs of the hiPSC-laden hydrogel, with well-defined line widths (~800 μm).

## 1. Introduction

Due to the increasing need for organ transplants and the limited number of organ donors, tissue engineering has made progress towards new technologies for tissue development [1], including cardiac muscle (myocardium) tissue. The myocardium can be subjected to a multitude of insults, such as myocardial infarction, infection, trauma, neoplasms, metabolic disorders and genetic diseases. Under these circumstances, which result in cardiac functional losses, the myocardium presents poor ability to naturally regenerate. One of the most promising regenerative strategies proposed in recent years aims to harness the ability of pluripotent stem cells (PSCs) to differentiate into all tissue types, including cardiac cells; indeed, their use in combination with new biomaterials, capable of emulating the behaviour of the extracellular matrix, represents a new frontier in the realm of cardiac regeneration [2,3].

As for any other tissue, engineering cardiac tissue requires advanced biomanufacturing strategies. At present, the ability of 3D printing to fabricate scaffolds capable of supporting cellular functions is being intensely explored. Conventional strategies typically involve seeding cells onto prefabricated scaffolds, which often results in random distributions of cells, unable to mirror the complex hierarchical organisation of functional tissues. Consequently, 3D printing of cell-laden bioinks, commonly referred to as bioprinting, is being investigated as a possible alternative [4].

A variety of bioinks for bioprinting have been developed with the aim of providing embedded cells with a viscoelastic and hydrated microenvironment capable of facilitating nutrient diffusion and extracellular matrix remodelling [5]. The selection of the bioink material is of paramount importance, as it dictates the physical and chemical properties of the final printed constructs [6,7,8,9].

Today, extrusion-based bioprinting is widely used with a diverse range of hydrogels, including natural polymers, such as collagen, gelatin, alginate, fibrin and hyaluronic acid [10,11,12,13,14,15], as well as synthetic polymers, such as poly(ethylene glycol) diacrylates and poloxamers [15,16]. Among these, naturally derived hydrogels are especially attractive, because they ensure the highest biocompatibility. In particular, gelatin functionalized with methacrylate groups (GelMA) has been extensively applied in extrudable bioink formulations [17]. Methacrylate-based modifications of gelatin typically have a dual advantage: they allow for harnessing the unique properties of gelatin, while simultaneously making the material capable of a permanent transition from the liquid to the solid state through chemical reaction of the methacrylate groups [18]. To this end, the material also requires the addition of a cytocompatible photoinitiator (e.g., Irgacure 2959 or LAP) and the excitation of such with ultra-violet (UV) light, which triggers a radical photopolymerisation reaction [19]. By varying the GelMA degree of methacrylation and/or the UV power density used for photo-curing, the polymer’s cross-linking density and thus its mechanical and biological properties can be varied [20]. Accordingly, methacrylated gelatin is widely applied in tissue regeneration [21].

To ensure the survival and proliferation of cells, a key role is played by the hydrogel stiffness, which can be adjusted to closely match the native tissue’s stiffness. Studies have reported that cardiac muscle tissue exhibits a stiffness of about 8–12 kPa [22,23], and GelMA can show elastic moduli of that order at concentrations between 5% and 10% *w*/*v* [24,25]. Moreover, GelMA has been reported to promote viability of a variety of cell types at concentrations up to ~5% *w*/*v* [26,27,28].

However, when GelMA has been used as a bioink at a concentration of 5% for bioprinting, its typically low viscosity has been shown to cause poor retention of shape after extrusion, i.e., poor printability [26,27,28]. Indeed, in general, the printability of a bioink is determined by its viscosity as a trade-off between opposite needs: on one hand, the viscosity should be sufficiently low to ensure ease of extrusion at low pressures, enabling a homogeneous distribution of the cells within the ink and protecting them from excessive shear stresses during the extrusion through sub-millimetric needles; on the other hand, the viscosity should also be sufficiently high to guarantee the preservation of the printed material shape after extrusion during the curing process.

Nevertheless, when GelMA has been used at concentrations of 10% *w*/*v* or higher to promote printability, printed structures have been shown to have excessively high stiffness and low porosity, limiting cell viability [27,28,29,30].

Therefore, in order to use GelMA hydrogels as bioinks, it is of paramount importance to balance the printability of the material and its biological functionality in terms of cell proliferation and viability. A suitable trade-off between these two requirements remains a challenge in 3D printing of GelMA bioinks [28].

To meet this need, it can be useful to combine GelMA with other polymers, such as xanthan gum, alginate or hyaluronic acid [31,32]. In particular, xanthan gum (XG) additions have recently been shown to favour increases in the bioink viscosity, ensuring retention of shape after extrusion. For example, Iervolino et al. reported that a bioink based on 7% *w*/*v* GelMA with the addition of 3% *w*/*v* XG was printable and able to preserve the shape of printed structures [7]. The biocompatibility of that formulation was investigated with indirect cytotoxicity tests by exposing L929 cells to a culture medium containing the hydrogel’s degradation products, which were found to be non-cytotoxic [7]. In the same year, Li et al. developed a novel GelMA/alginate-based composite hydrogel, incorporating polyethylene glycol dimethacrylate and XG to improve printability and control the mechanical properties of the final construct [31]; bioprinting tests with mesenchymal stem cells demonstrated the ability of the bioink to support cell viability [31]. While those studies provided preliminary evidence that combining GelMA with XG was able to generate a stable bioink that supported the viability of certain types of cells, the resulting hydrogels exhibited elastic moduli higher than 14 kPa [7,31], which are too high for cardiac tissue engineering.

According to the present state of the art, the aim of this study was to develop a 3D-printable GelMA-based hydrogel bioink satisfying the following two requirements: (i) it should enable the biofabrication of constructs exhibiting an elastic modulus comparable to that of cardiac tissue; (ii) it should preserve the viability and proliferation of human induced pluripotent stem cells (hiPSCs), so as to then promote their differentiation into cardiomyocytes within the hydrogel matrix for cardiac tissue engineering.

To that end, GelMA was firstly prepared at a concentration of 5% *w*/*v* and then was combined with XG. The composite bioink was characterised in terms of mechanical properties and 3D printability, as well as proliferation of hiPSCs and their differentiation into cardiac cells.

## 2. Materials and Methods

### 2.1. Hydrogel Preparation

The GelMA hydrogel was prepared as follows. Lyophilized GelMA (60% degree of substitution, Sigma Aldrich, St. Louis, MO, USA) was firstly dissolved in either a phosphate-buffered saline solution (PBS 1Χ, pH 7.4, by Gibco, Thermofisher Scientific, Waltham, MA, USA) or a sterile cell culture medium (mTeSR, pH 7.5, by Stem Cell Technologies, Vancouver, Canada) depending on the specific test, as detailed in subsequent sections. The dissolution was performed at a concentration of 5% *w*/*v* and at a temperature of 40 °C under magnetic stirring until complete dissolution. Then, a photoinitiator (Lithium phenyl-2,4,6-trimethylbenzoylphosphinate—LAP, Sigma Aldrich, St. Louis, MO, USA) was added to the GelMA solution at a concentration of 0.25% *w*/*v*.

The GelMA-XG composite hydrogel was prepared by incorporating xanthan gum (Sigma Aldrich, St. Louis, MO, USA) into the GelMA solution at a concentration of 1.25% *w*/*v* and at a temperature of 40 °C under magnetic stirring. Then, the LAP photoinitiator was added to the GelMA-XG solution at a concentration of 0.25% *w*/*v*.

Each type of hydrogel was finally obtained by cross-linking the solution under UV light at a wavelength of 405 nm, generated by the UV LED source of the bioprinter used in this work (Cellink, Goteborg, Sweden—as described in the following). The LED source was used at its maximum intensity (default setting), which was measured (with an optical power meter) and was found to correspond to a power density of 33 mW/cm^2^.

The duration of cross-linking varied depending on the thickness of the sample to be cured. According to conventional practice and Cellink protocols, we adopted a cross-linking time of approximately 10 s for every 1 mm of specimen thickness.

### 2.2. Bioink Preparation

The GelMA-XG solution was also used to prepare a bioink, by mixing it with hiPSCs at varying concentrations, depending on the specific test (as detailed in the subsequent sections). Sterile conditions were ensured by standard procedures (washing with ethanol and UV sterilisation) and a continuous laminar flow.

The bioink was cross-linked at 405 nm (with an optical power density of 33 mW/cm^2^) for a duration that depended on the sample thickness, as indicated above.

### 2.3. Swelling Test

In order to investigate the swelling behaviour, swelling tests were performed on samples of the GelMA and GelMA-XG hydrogels, shaped as 3 mm thick flat specimens, obtained by casting the material into a polydimethylsiloxane (PDMS) mould. All samples were crosslinked at 405 nm (with an optical power density of 33 mW/cm^2^) for 30 s per side.

The swelling rate of the two hydrogels was studied by incubating the samples at 25 °C in PBS 1Χ and weighing the swollen samples at fixed times, until they reached an equilibrium, i.e., a stabilisation of their weight. In particular, the swelling rate was monitored as the time evolution of the swelling ratio, defined as follows:

(1) $$Swelling\;ratio=\frac{w_{s,t\;}-w_{d}}{w_{d}}\;$$

where *w_s,t_* is the weight of the swollen sample at time *t* and *w_d_* is the initial weight of the dried sample.

### 2.4. Quasi-Static Compression Test

Indentation tests were performed to characterise the hydrogels’ quasi-static stiffness under compression, which represents a key property for the survival and proliferation of cells within a soft material that mimics the extracellular matrix.

For each type of hydrogel (GelMA and GelMA-XG), 3 mm thick flat specimens were obtained by mould casting. The samples were cross-linked at 405 nm (with an optical power density of 33 mW/cm^2^) for 30 s per side. Their stiffness was determined in a fully hydrated state. To that end, the samples were firstly immersed in PBS 1Χ at room temperature until they reached swelling equilibrium (i.e., a stabilized weight).

Then, they were subjected to indentation tests using a custom-built system consisting of a flat cylindrical indenter (diameter of 5 mm) connected to a load cell (HALJIA HX711) mounted on a micrometric three-axial translation stage (product code 7400774616774, Temkin, Waban, MA, USA). Indentation steps equal to 5% of the sample thickness were progressively applied, up to a maximum indentation of 50%. At each step, the force exerted by the specimen in response to the applied indentation was recorded and the related stress was calculated, thereby generating a stress–indentation curve.

### 2.5. Degradation Test

In tissue engineering, cell scaffold materials typically need to possess an appropriately tuned degradation rate. In particular, the material should degrade fast enough to leave space for cells to deposit new extracellular matrix; however, the degradation should not be excessively rapid to preserve the mechanical integrity of the printed constructs [7]. Therefore, a degradation test was performed on the GelMA and GelMA-XG hydrogels as follows.

For each hydrogel, 3 mm thick specimens were obtained by mould casting, and were cross-linked at 405 nm (with an optical power density of 33 mW/cm^2^) for 30 s per side. The test consisted of maintaining the samples immersed in PBS 1Χ (+0.1% penicillin/streptomycin (P/S)) at 37 °C for 28 days and measuring the progressive reduction in weight over time due to the degradation. The first measurement was taken at 24 h from the beginning of the test in order to ensure that the samples had reached their swelling equilibrium (according to the results of preceding swelling tests, reported in the following). Prior to each measurement, the PBS 1Χ solution was removed, and after the measurement, a new one was added.

The degradation rate was monitored as the time evolution of the degradation ratio, defined as follows:

(2) $$Degradation\;ratio=\frac{w_{s,e\;}-w_{deg,\;t}}{w_{s,e}}$$

where *w_s,e_* is the initial weight of the swollen hydrogel at equilibrium and *w_deg,t_* is the weight at time *t* of the degraded hydrogel.

### 2.6. Printability Tests

Prior to any investigation on the suitability of the GelMA-XG hydrogel as a bioink, its 3D printability was assessed with extrusion tests, aimed at a reaching a trade-off between ability of extrusion through a sub-millimetre needle and shape retention after printing.

To that end, an extrusion-type bioprinter (BIOX6, Cellink, Goteborg, Sweden) was used to create several horizontal lines by extruding the GelMA-XG hydrogel through a 22G conical needle. A variety of extrusion conditions were experimented with by varying the extrusion temperature at the needle (20 to 26 °C), the extrusion pressure (15 to 25 kPa) and the extrusion speed (5 to 6 mm/s). A suitable trade-off between extrudability and shape retention was empirically achieved with the following printing parameters, identified with trial-and-errors tests: extrusion temperature: 22.5 °C; extrusion pressure: 17 kPa; extrusion speed: 5 mm/s. The samples were cross-linked at 405 nm (with an optical power density of 33 mW/cm^2^) for 15 s on top of a temperature control plate maintained at 15 °C.

Sterile conditions were ensured by a 5 min UV sterilisation of the printing chamber prior to printing and a continuous laminar flow during printing.

The quality of the extrudable constructs (determined by the composition of the hydrogel and its printing parameters) was evaluated by quantifying the following two frequently used variables: ‘printing accuracy’ and ‘printability’.

The printing accuracy was evaluated by comparing the nominal area of a geometric shape to be printed and the actual area of the printed version. According to conventional practice, the employed shape was a square frame. In particular, a 12 × 12 mm square frame with a frame width of 0.41 mm (corresponding to the inner diameter of the extrusion needle) was printed, photographed and analysed (with the ImageJ 1.54i software) to calculate the printed area. The printing accuracy was quantified according to the following formula [33]:

(3) $$Printing\;accuracy=1-\frac{\;\left|A_{p\;\;\;}-A_{n\;\;\;}\right|}{A_{n\;\;\;}}$$

where *A_n_* is the nominal area of the frame and *A_p_* is the area of its printed version. The printing accuracy was averaged among five specimens of the frame.

The printability was evaluated, according to conventional practice, by printing a 4 × 4 grid of square pores and quantifying for each pore *i* the following variable [34]:

(4) $$Printability=\frac{{P_{i\;\;\;}}^{2}}{16\;A_{i\;\;\;}}$$

where *P_i_* and *A_i_* are the inner perimeter and inner area, respectively, of the pore. It is worth noting that for a perfect square pore the printability would be 1, while for imperfect printed structures the printability may be higher or lower than 1. In our tests, the grid had a size of 12 × 12 mm with a line width of 0.41 mm (as for the printing accuracy test). The printability was averaged among multiple pores of five specimens of the grid.

### 2.7. HiPSCs Preparation

Undifferentiated hiPSCs (cell line AICS-0037 cl.172, Allen Institute for Cell Science, Seattle, WA, USA) were received as a gift (Dr. Albano Meli, CRCN INSERM, Montpellier, France). In the hiPSC clonal line, the gene TNNI1 had endogenously been tagged (by using the CRISPR/Cas9 technology) with the monomeric Enhanced Green Fluorescent Protein (mEGFP), which can be used to track the expression of the slow skeletal Troponin I (ssTnI) sarcomere protein as an indicator of cardiomyocyte differentiation. Indeed, ssTnI is expressed early during any process of cardiac differentiation, including that which leads to hiPSC cardiomyocytes. In particular, as during the early phases of cardiac induction the ssTnI is expressed together with mEGFP, the fluorescence of the latter was used as a marker of successful cardiac differentiation.

Colonies of the hiPSCs were expanded in mTeSR with +0.1% penicillin/streptomycin (P/S) (Thermo Fisher Scientific, Waltham, MA, USA) on a Matrigel matrix (Corning Incorporated, Corning, NY, USA), at 37 °C and 5% CO_2_. Upon reaching a confluency of 70–80%, the colonies were chemically dissociated by using Tryple 1X (Thermo Fisher Scientific, Waltham, MA, USA) and incubated at 37 °C for approximately 3–5 min. The cell suspension was centrifuged at 200 rpm for 5 min at room temperature and the resulting pellet was resuspended in 1 mL of mTesR (+0.1% P/S) with 5 μM of ROCK inhibitor (Stem Cell Technologies, Vancouver, Canada). The cells were finally used for cell seeding onto hydrogel layers or resuspension into the hydrogel solution, depending on the test to be performed, as described in the following.

### 2.8. HiPSCs Proliferation Test on 2D Cultures

The ability of hiPSCs to proliferate within 2D cell cultures on top of layers of the GelMA-XG and GelMA hydrogels was evaluated with preliminary tests. Moreover, to investigate whether the proliferation could be improved with an extracellular matrix protein, the two hydrogel formulations were also supplemented with fibronectin (FN) for comparison according to previous studies [2].

For these tests, the GelMA and GelMA-XG hydrogels were prepared according to the procedure described in the previous sections, with the only difference being that the dissolution of GelMA was performed within a culture medium (mTeSR, Stem Cell Technologies, Vancouver, Canada) (+0.1% P/S) instead of PBS 1Χ. To create the new GelMA-XG-FN and GelMA-FN hydrogels, 50 µg/mL of FN (Corning Incorporated, Corning, NY, USA) was added to each formulation.

Hydrogel layers (1–2 mm thick) were cross-linked at 405 nm (with an optical power density of 33 mW/cm^2^) for 15 s. The layers’ surface was then seeded with hiPSCs (~2 × 10^3^ cells/mm^2^) following their chemical dissociation and resuspension within the culture medium (mTeSR, Stem Cell Technologies, Vancouver, Canada) (+0.1% P/S), which was complemented with a ROCK Inhibitor (RI) (Stem Cell Technologies, Vancouver, Canada) at a concentration of 5 μM.

The specimens were kept in culture at 37 °C and 0.5% CO_2_ up to 9 days and were observed with an optical microscope (EVOS FL auto 2, Thermo Fisher Scientific Inc., Waltham, MA, USA).

### 2.9. HiPSCs Proliferation Tests within 3D Cultures

To assess the ability of hiPSCs to proliferate within 3D cell cultures inside the GelMA-XG and GelMA-XG-FN hydrogels, the following test was performed.

The GelMA-XG and GelMA-XG-FN hydrogels were prepared using the same procedure described for the 2D cell cultures tests. HiPSCs were chemically dissociated and resuspended in the mTeSR culture medium (+0.1% P/S), which was complemented with 5 μM RI. The cells were added to the GelMA-XG and GelMA-XG-FN solutions at a concentration of ~2.3 × 10^3^ cells/mL.

Then, small droplets (5–10 μL in volume) of the bioink were pipetted into a multiwell plate and were cross-linked at 405 nm (with an optical power density of 33 mW/cm^2^) for 15 s. Moreover, to investigate the effect of the temperature of the substrate above which the hydrogels where cross-liked, the cross-linking was performed on top of a temperature control plate, which was used to apply different temperatures to different samples: 25 °C or 15 °C. This was aimed at assessing the role that the temperature of the printing bed would have during a 3D printing and curing process. Therefore, the temperature control plate adopted for this test coincided with the heated print bed of the bioprinter.

Subsequently, the so-created cell-laden hydrogel hemispheres were cultured in the mTeSR culture medium (+0.1% P/S) with the addition of RI at a concentration of 5 μM at 37 °C and 5% CO_2_ for 7 days. Optical microscope images (Olympus CKX53, Evident Corporation, Tokyo, Japan) were acquired at regular intervals.

### 2.10. HiPSCs Cardiac Differentiation Tests within 3D Cultures

On day 7 of culturing of the cell-laden GelMA-XG hydrogel hemispheres, the cell differentiation process was initiated using the PSC Cardiomyocyte Differentiation Kit (Thermo Fisher Scientific Inc., Waltham, MA, USA). In particular, the process started by adding the differentiation medium A of the kit. This corresponded with day 0 of the differentiation protocol. After two days, medium A was replaced with the differentiation medium B, which was subsequently replaced, after two days, with the maintenance medium C. Then, the maintenance medium C was changed every two days. From day 12 of cellular maturation, the samples were maintained in the RPMI/B27 medium (Thermo Fisher Scientific Inc., Waltham, MA, USA) (+0.1% P/S).

Subsequently, images were acquired using a fluorescence microscope (EVOS FL auto 2, Thermo Fisher Scientific Inc., Waltham, MA, USA). This allowed for the visualisation of the fluorescence emission by the mEGFP-labelled slow skeletal Troponin I, which was expressed only by the cells that had been differentiated into cardiomyocytes.

Additionally, confocal microscopy tests were performed to analyse the action potentials generated by the matured cardiomyocytes. The samples were stained with 1 μL/mL of an electrically sensitive fluorescent dye (Di-8-ANEPPS, Thermo Fisher, Waltham, MA, USA), which was used as an optical probe of the cell membrane potential. The dye was added to the Tyrode experimental solution (5 mM HEPES, 10 mM Glucose, 140 mM NaC1, 5.4 mM KCI, 1.2 mM MgCl_2_, mM 1.8 CaCl_2_, pH = 7.3) inside an experimental chamber. The chamber was equipped with platinum electrodes used to deliver controlled electrical stimulation. The latter consisted of repeated voltage pulse trains (90 V pulse amplitude, for an electrode separation of 1 cm) generated by an external stimulator (DigiTimer, Welwyn Garden City, UK). Each train consisted of a ~50 ms sequence of pulses, each having a duration (pulse width) of 0.08 ms. The pulse trains were repeated at a frequency of either 0.5 or 1 Hz for the sake of comparison.

During the recordings, the samples were continuously perfused with Tyrode solution heated to 37 °C. A blue LED light (467 nm) was used for fluorescence studies to excite the dye in the samples. The emitted fluorescence was collected by a camera (Photometrics Prime sCOS Teledyne, Tucson, AZ, USA) and processed through a dual-wavelength bandpass filter cube (IDEX, Semrock, West Henrietta, NY, USA). The filter allowed for collecting orange radiation (631 nm) corresponding to the emission of the dye. The fluorescence images were analysed using a proprietary software (MetaMorph, Molecular Devices, San Jose, CA, USA). Data related to various regions of interest were acquired from each sample.

### 2.11. HiPSCs Bioprinting Test

In order to perform preliminary bioprinting tests, the GelMA-XG hydrogel was prepared using the same procedure described for the 2D and 3D cell cultures tests. HiPSCs were chemically dissociated and resuspended in the mTeSR culture medium (+0.1% P/S), which was complemented with 5 μM RI. To create the bioink, the cells were added to the GelMA-XG solution, at a concentration of ~1.3 × 10^3^ cells/mL. Once the bioink was balanced at 37 °C, it was transferred to the print cartridge, which was then mounted on the bioprinter (BIOX6, Cellink, Goteborg, Sweden).

Test lines were 3D printed with the bioink and cross-linked at 405 nm (with an optical power density of 33 mW/cm^2^) for 15 s.

### 2.12. Statistical Analysis

Quantitative results were expressed as mean values ± standard deviation. In consideration of the small number of samples available for each type of measurement, it was not possible to verify the data distribution’s normality. Therefore, non-parametric statistical analyses were performed, using the Wilcoxon–Mann–Whitney test. The test was implemented within the software R (R Core Team, Vienna, Austria), using the function ‘wilcox.test()’. Differences were considered statistically significant for *p* < 0.05.

## 3. Results and Discussion

### 3.1. Hydrogel Swelling

**Figure 1:**
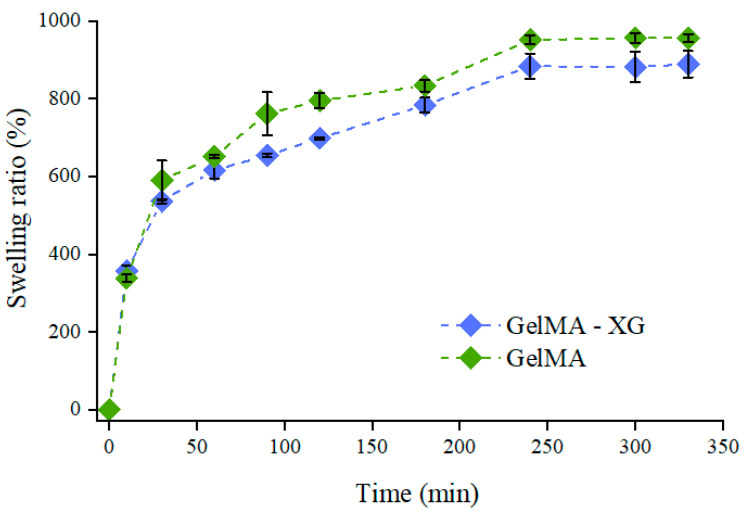
Swelling ratio as a function of time for the GelMA and GelMA-XG hydrogels. The error bars represent the standard deviation among four samples. A Wilcoxon–Mann–Whitney test indicated a statistically non-significant difference between the GelMA and GelMA-XG series of data (*p* = 0.2975).

Figure 1 presents the swelling dynamics of the GelMA and GelMA-XG hydrogels. The swelling ratio increased over time until equilibrium, which was reached after about 5 h.

These swelling tests indicated that the hydrogel’s ability to absorb water was not significantly affected by the presence of XG. Indeed, while the swelling ratio at equilibrium appeared slightly higher for GelMA (Figure 1), no statistically significant difference was obtained relative to GelMa-XG (*p* > 0.05). This likely occurred because of two concomitant antagonist effects induced by the addition of XG: on one hand, it created an interpenetrating polymer network, which tended to increase the material’s density and stiffness, as also confirmed by the results of the indentation tests (presented below); however, on the other hand, it also decreased the cross-link density (because of a reduction in the density of methacrylate groups), thereby facilitating the GelMA swelling.

### 3.2. Hydrogel Stiffness

**Figure 2:**
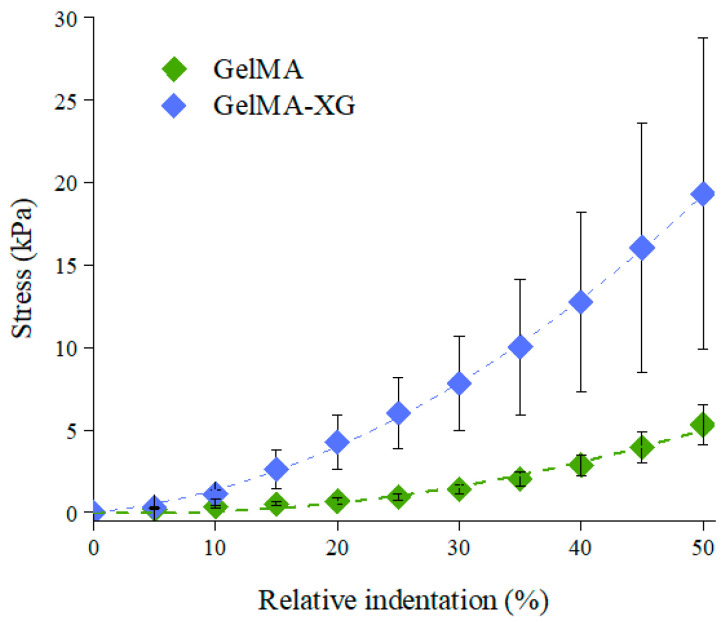
Stress-relative indentation curves of the GelMA and GelMA-XG hydrogels. The error bars represent the standard deviation among four samples. A Wilcoxon–Mann–Whitney test indicated a statistically significant difference between the GelMA and GelMA-XG series of data (*p* = 0.0001942).

The characterisation of the quasi-static stiffness of the GelMA and GelMA-XG hydrogels under compression is presented in Figure 2, which plots the compressive stress as a function of the relative indentation (defined as the ratio between the absolute value of the reduction in thickness of the sample and its thickness at rest).

The compressive elastic modulus of each hydrogel was estimated as the initial slope of a second-order fitting curve for the data. The GelMA hydrogel exhibited an elastic modulus of ~4 kPa, which was consistent with findings reported in the literature [35].

However, the addition of XG led to an increase in stiffness, resulting in a compressive elastic modulus of ~9 kPa. This value was comparable with those reported for the cardiac muscle tissue (8–12 kPa) [22,23]. This indicates that the GelMA-XG hydrogel can adequately match the mechanical properties of the biological tissue of interest.

### 3.3. Hydrogel Degradation

**Figure 3:**
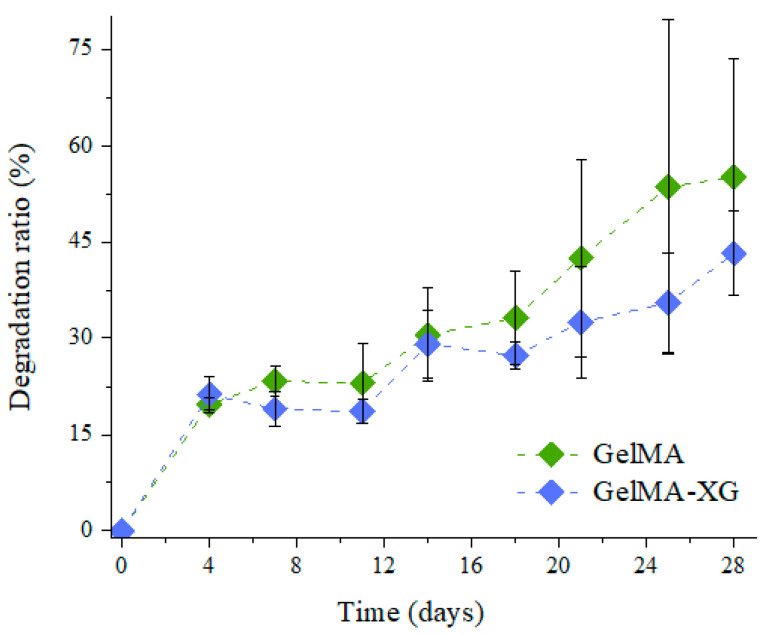
Degradation ratio as a function of time for the GelMA and GelMA-XG hydrogels. The error bars represent the standard deviation among two samples. A Wilcoxon–Mann–Whitney test indicated a statistically non-significant difference between the GelMA and GelMA-XG series of data (*p* = 0.3624).

The degradation ratio of the GelMA and GelMA-XG hydrogels as a function of time is shown in Figure 3.

As compared to the GelMA hydrogel, the GelMA-XG composite did not exhibit statistically significant differences in terms of degradation. Indeed, while the degradation ratio appeared as slightly higher for GelMA (Figure 3), no statistically significant difference was obtained relative to GelMA-XG (*p* > 0.05). Such an outcome was consistent with the results of the swelling test (Figure 1).

### 3.4. Hydrogel Printability

**Figure 4:**
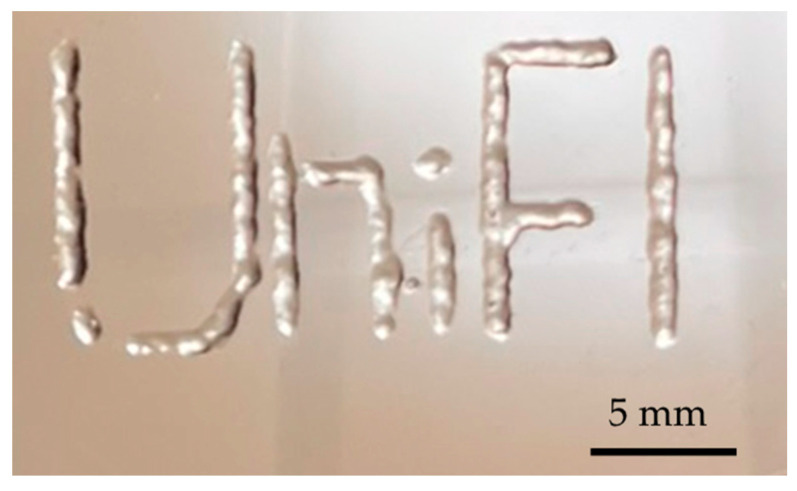
Example of a structure 3D printed with the GelMA-XG hydrogel.

As a qualitative example of the printability of the GelMA-XG hydrogel, Figure 4 presents a 3D-printed structure.

**Figure 5:**
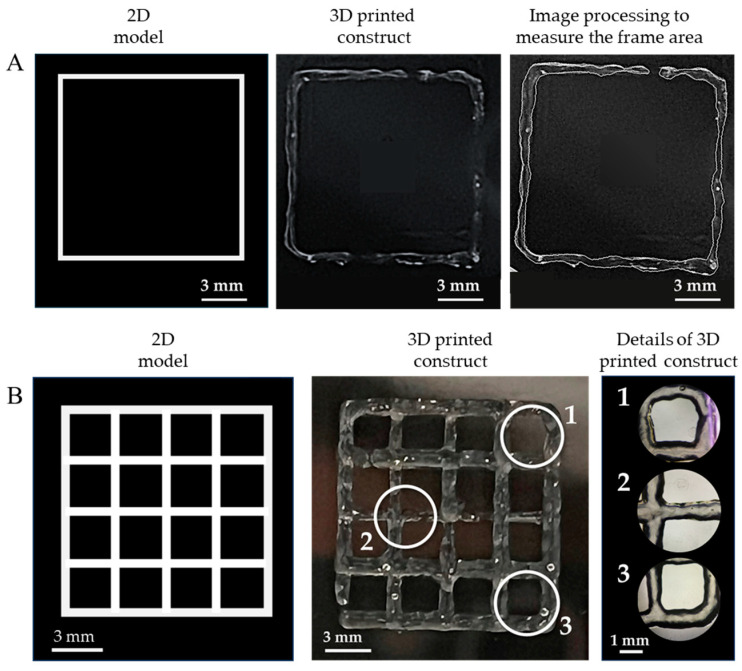
Assessment of the quality of the extrudable constructs made of the GelMA-XG hydrogel: (**A**) square frame and example of a 3D-printed version, used to quantify the printing accuracy; (**B**) grid of square pores and example of a 3D-printed version, used to quantify the printability; zoomed-in views on the right-hand side panel show detailed areas (1, 2, 3) of the printed grid.

As an assessment of the quality of the extrudable constructs, Figure 5 presents the geometric shapes that were 3D printed with the GelMA-XG hydrogel to quantify the ‘printing accuracy’ and ‘printability’.

In particular, Figure 5A shows an example of a 3D-printed square, which was used to measure the printing accuracy. By taking measurements from five specimens, the average printing accuracy was 83% (with an error of ±9%). This outcome, which was consistent with findings from other studies on 3D printing of gelatin-based bioinks [33,36], indicates that the combined hydrogel and printing process enabled the fabrication of structures with rather good accuracy, due to an ability to retain shape after extrusion.

Figure 5B presents an example of a 3D-printed grid used to measure the printability. By taking measurements from five specimens, the average printability was 0.98 (with an error of ±0.02). This number was not only included within the range of values (from 0.9 to 1.1) that are typically regarded as indicative of good printing properties [34], but also very close to the ideal case (printability = 1). This demonstrates the high printability of the GelMA-XG hydrogel.

For the sake of comparisons, it is worth noting that the achieved printability was higher than the values (from 0.89 to 0.91) previously reported for GelMA at a concentration of 10% *w*/*v* [4]. Therefore, this shows that the creation of a composite between GelMA at a concentration of 5% *w*/*v* and XG was more effective, in terms of printability, than simply increasing the GelMA concentration to 10% *w*/*v* (without any addition of XG).

### 3.5. HiPSCs Proliferation on 2D Cultures

**Figure 6:**
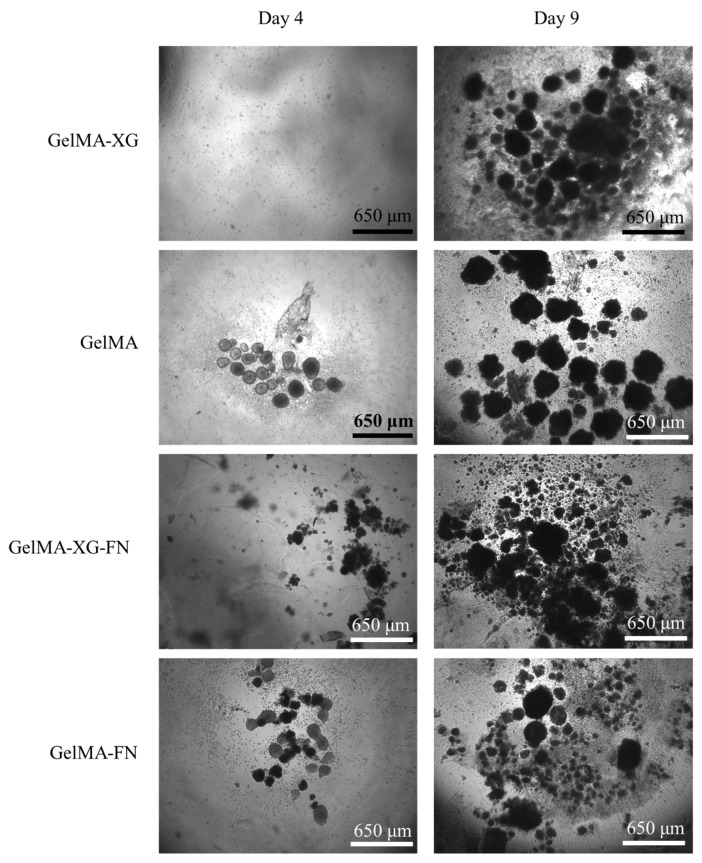
Optical microscopy images of hiPSCs within 2D cell cultures on top of layers of the GelMA-XG, GelMA, GelMA-XG-FN and GelMA-FN hydrogels, taken after 4 and 9 days from seeding.

The results of a preliminary test of hiPSC proliferation on the GelMA-XG, GelMA, GelMA-XG-FN and GelMA-FN hydrogels are presented in Figure 6.

From day 4 to day 9, the number and size of cell colonies increased for all materials, indicating that all of them enabled the proliferation of the cells.

The addition of fibronectin to the formulations was not found to lead to evident significant differences in terms of cell proliferation, as shown by the images in Figure 6.

### 3.6. HiPSC Proliferation within 3D Cultures

**Figure 7:**
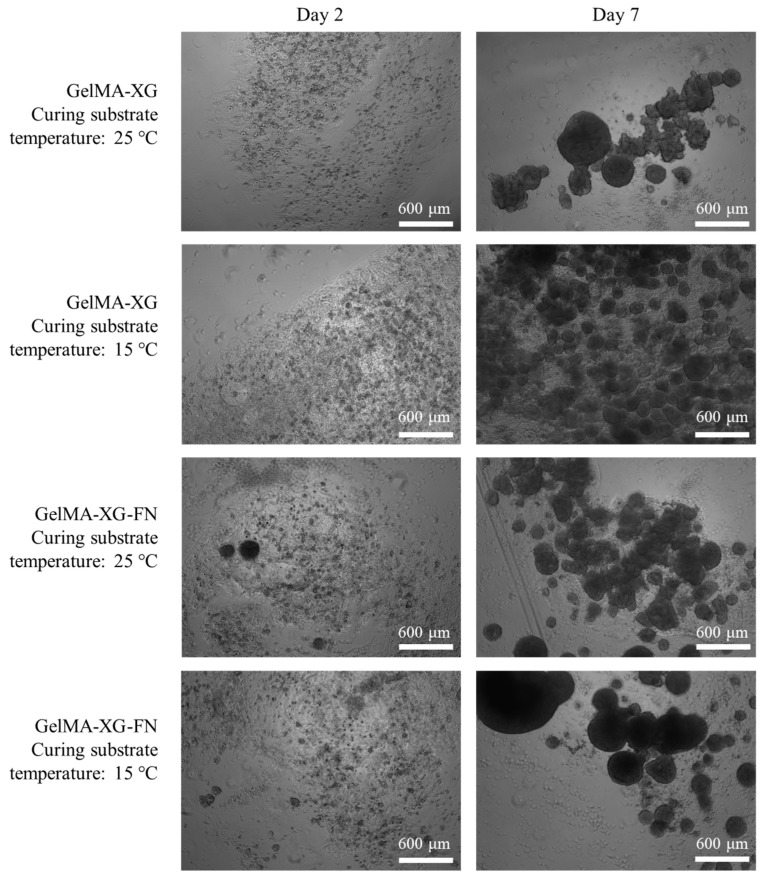
Optical microscopy images of hiPSCs within 3D cell cultures inside hydrogel hemispheres made of GelMA-XG and GelMA-XG-FN. The images were taken after 2 and 7 days from UV cross-linking for 15 s, which occurred above a substrate maintained at a temperature of 25 °C or 15 °C.

The hiPSC proliferation within 3D cell cultures inside the GelMA-XG and GelMA-XG-FN hydrogel hemispheres is presented in Figure 7.

The images show that both hydrogels facilitated the cellular proliferation. As for the 2D cell culture tests, even in these tests the addition of fibronectin was not found to cause evident differences in terms of cellular proliferation.

Figure 7 also presents the effect of a variation of the temperature of the substrate above which the hydrogels were cross-linked (by UV irradiation). This provides preliminary information, which might be useful to plan future bioprinting strategies. Indeed, they might take advantage of cooling the 3D printing bed to reduce the viscosity of the extruded material and facilitate the retention of shape during cross-linking. The results in Figure 7 show that cooling the substrate down to 15 °C did not apparently limit the ability of the cells to proliferate. Nevertheless, such preliminary evidence provides initial indications that necessarily cannot be taken as conclusive, as they should be confirmed with future systematic studies.

### 3.7. HiPSC Cardiac Differentiation within 3D Cultures

Eight days after the initiation of the differentiation process within the cell-laden GelMA-XG and GelMA-XG-FN hydrogel hemispheres, the cells exhibited significant spontaneous contraction, which revealed their differentiation into cardiomyocytes. Videos showing contractions of the differentiated cells are available in the Supplementary Materials.

**Figure 8:**
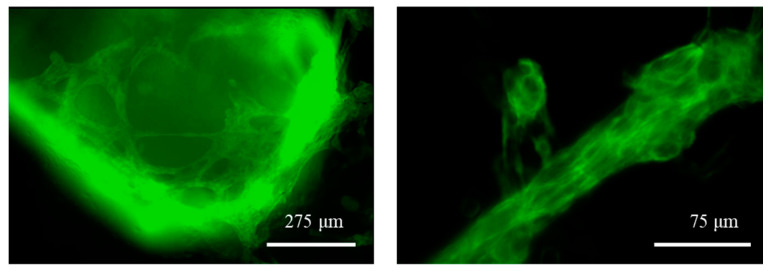
Fluorescence microscopy images of the GelMA-XG hydrogel hemispheres containing differentiated hiPSC cardiomyocytes at day 8 of the differentiation process. The differentiation is indicated by the endogenous expression of the mEGFP fluorescent marker. See the videos in the Supplementary Materials which show specimens contracting at a frequency of ~0.8 Hz (highest value recorded).

The differentiation was also evidenced by the fluorescence caused by the expression of the mEGFP-labelled slow skeletal Troponin I in the cell sarcomeres, as shown in Figure 8. The fluorescence was used a reliable indicator, as hiPSC cardiomyocytes are well known to express, early after cardiac differentiation, that type of Troponin [37], which is therefore widely used as a cardiac maturation marker of hiPSC cardiomyocytes.

The frequency and amplitude of contraction were measured by processing the videos (with the software ImageJ). On day 8 from the beginning of the differentiation process, different specimens of the GelMA-XG and GelMA-XG-FN hydrogel hemispheres were found to exhibit an average contraction frequency of 0.5 Hz (±0.2 Hz standard deviation) and 0.3 Hz (±0.1 Hz standard deviation), respectively, while their average contraction amplitude was 2.0% (±1.5% standard deviation) and 1.1% (±1.4% standard deviation), respectively. Such values of frequency and amplitude were consistent with those of immature cardiomyocytes [38]. This was likely due to the structural/functional immaturity of the cardiomyocytes (on day 8 from the beginning of the differentiation), as well as the fact that the recordings took place at room temperature (~23 °C).

**Figure 9:**
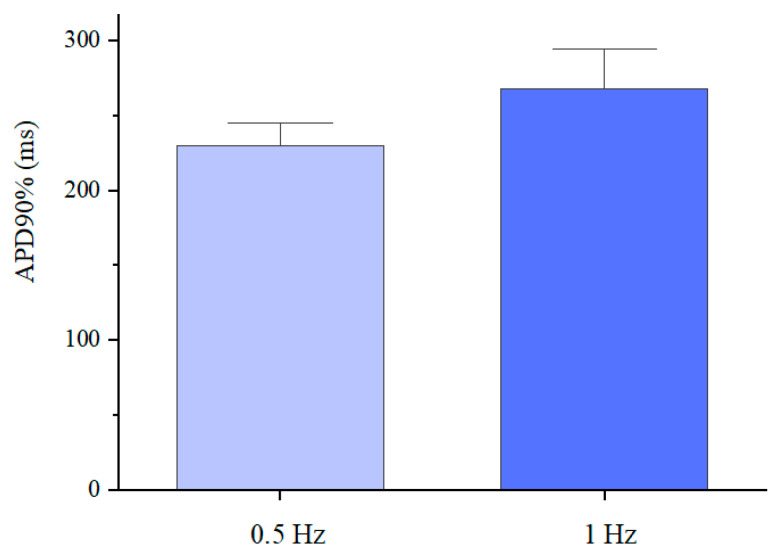
Action potential duration at 90% repolarisation of the differentiated cardiomyocytes inside the GelMA-XG hydrogel hemispheres, on day 17 of the differentiation process, in response to an electrical stimulation at 0.5 Hz or 1 Hz, at 37 °C. The error bars represent the standard deviation among three samples.

The differentiated cardiomyocytes were also subjected to a fluorescence-based optical analysis of action potentials induced by electrical stimulation (as detailed in the Materials and Methods section). In particular, the analysis was used to determine the action potential duration at 90% repolarisation (APD90%), the values of which are presented in Figure 9.

The average values (230 and 268 ms) were consistent with those reported in the literature for single hiPSC cardiomyocytes [39,40], although they were lower than typical values (~350 ms) of mature cardiomyocytes [41]. These results indicated that the electro-physiological phenotype of the differentiated cardiomyocytes was distinct from that of adult cardiomyocytes, due to different expressions of ionic channels, owing to the cardiomyocytes’ immaturity [42,43].

### 3.8. HiPSCs Bioprinting

**Figure 10:**
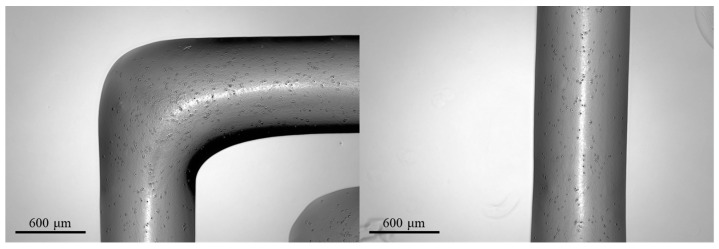
Optical microscopy images of 3D-printed structures made of the hiPSC-laden GelMA-XG hydrogel.

The results of a preliminary test of bioprinting of the hiPSC-laden GelMA-XG bioink are presented in Figure 10.

The 3D-printed structures showed a high retention of shape, with well-defined edges and a line width of ~800 μm.

However, the structures were found to display a low cell density, as visible in the images; this might straightforwardly be addressed in the future by increasing the cell concentration in the bioink above the value used in these preliminary tests (~1.3 × 10^3^ cells/mL).

## 4. Conclusions and Future Developments

We described the development of a bioink formulation for bioprinting of human induced pluripotent stem cells using a GelMA-based hydrogel.

Although previous studies had shown that GelMA at a low concentration (5% *w*/*v*) allowed the growth of a diversity of cells, GelMA was also known to limit the 3D printability of structures, owing to a poor retention of shape after extrusion caused by a low viscosity. To solve that problem, ensuring both a good printability of the bioink and high proliferations of stem cells, in this work a 3D-printable GelMA-based bioink was developed with the addition of XG.

The GelMA-XG hydrogel was shown to have a compressive elastic modulus (~9 kPa) comparable to that of cardiac tissue, as well as enable 3D printing, being characterised by high values of printing accuracy (83%) and printability (0.98).

Tests with human induced pluripotent stem cells showed the hydrogel’s ability to promote their proliferation within both 2D and 3D cell cultures. The tests also showed that stem cells inside hemispheres of the hydrogel were able to differentiate into colonies of cardiomyocytes, which displayed spontaneous contractile activity (average contraction frequency of ~0.5 Hz and amplitude of ~2%).

Preliminary bioprinting tests demonstrated the possibility of extruding the cell-laden hydrogel, producing structures with well-defined line widths (~800 μm).

Future studies might extend this preliminary work by performing more systematic investigations. In particular, the number of samples to be tested for each type of characterisation should be increased to enable meaningful statistical analyses. Furthermore, the tests might be complemented with additional ones, such as the measurement of the materials’ rheological and morphological properties. Nevertheless, the most important goal should be assessing how 3D structures that will be printed using the new bioink will behave in terms of stem cell viability, proliferation and differentiation.

## Data Availability

The data that support the findings of this study are available from the corresponding author upon reasonable request.

## Supplementary Materials

The following supporting information can be downloaded at: https://www.mdpi.com/article/10.3390/jfb15100297/s1: Video S1, Fluorescence microscopy of the spontaneous contraction of differentiated hiPSC-cardiomyocytes inside GelMA-XG hydrogel hemispheres; Video S2, Optical microscopy of the spontaneous contraction of differentiated hiPSC-cardiomyocytes inside GelMA-XG hydrogel hemispheres.

## References

[1] Zorlutuna P., Annabi N., Camci-Unal G., Nikkhah M., Cha J.M., Nichol J.W., Manbachi A., Bae H., Chen S., Khademhosseini A. (2012). Microfabricated biomaterials for engineering 3D tissues. Adv. Mater..

[2] Kupfer M.E., Lin W.H., Ravikumar V., Qiu K., Wang L., Gao L., Bhuiyan D.B., Lenz M., Ai J., Mahutga R.R. (2020). In situ expansion, differentiation, and electromechanical coupling of human cardiac muscle in a 3D bioprinted, chambered organoid. Circ. Res..

[3] MacQueen L.A., Sheehy S.P., Chantre C.O., Zimmerman J.F., Pasqualini F.S., Liu X., Goss J.A., Campbell P.H., Gonzalez G.M., Park S.J. (2018). A tissue-engineered scale model of the heart ventricle. Nat. Biomed. Eng..

[4] Jain T., Baker H.B., Gipsov A., Fisher J.P., Joy A., Kaplan D.S., Isayeva I. (2021). Impact of cell density on the bioprinting of gelatin methacrylate (GelMA) bioinks. Bioprinting.

[5] Kim J.S., Hong S., Hwang C. (2016). Bio-ink materials for 3D bio-printing. J. Int. Soc. Simul. Surg..

[6] Fan C., Wang D.A. (2017). Macroporous hydrogel scaffolds for three-dimensional cell culture and tissue engineering. Tissue Eng. Part B Rev..

[7] Iervolino F., Belgio B., Bonessa A., Potere F., Suriano R., Boschetti F., Mantero S., Levi M. (2023). Versatile and non-cytotoxic GelMA-xanthan gum biomaterial ink for extrusion-based 3D bioprinting. Bioprinting.

[8] Gao Q., Kim B.S., Gao G. (2021). Advanced strategies for 3D bioprinting of tissue and organ analogs using alginate hydrogel bioinks. Mar. Drugs.

[9] Fatimi A., Okoro O.V., Podstawczyk D., Siminska-Stanny J., Shavandi A. (2022). Natural Hydrogel-Based Bio-Inks for 3D Bioprinting in Tissue Engineering: A Review. Gels.

[10] Liu W., Heinrich M.A., Zhou Y., Akpek A., Hu N., Liu X., Guan X., Zhong Z., Jin X., Khademhosseini A. (2017). Extrusion bioprinting of shear-thinning gelatin methacryloyl bioinks. Adv. Healthc. Mater..

[11] Skardal A., Zhang J., Prestwich G.D. (2010). Bioprinting vessel-like constructs using hyaluronan hydrogels crosslinked with tetrahedral polyethylene glycol tetracrylates. Biomaterials.

[12] Schöneberg J., De Lorenzi F., Theek B., Blaeser A., Rommel D., Kuehne A.J.C., Kießling F., Fischer H. (2018). Engineering biofunctional in vitro vessel models using a multilayer bioprinting technique. Sci. Rep..

[13] Leucht A., Volz A.C., Rogal J., Borchers K., Kluger P.J. (2020). Advanced gelatin-based vascularization bioinks for extrusion-based bioprinting of vascularized bone equivalents. Sci. Rep..

[14] Shah P.P., Shah H.B., Maniar K.K., Özel T. (2020). Extrusion-based 3D bioprinting of alginate-based tissue constructs. Proc. CIRP.

[15] Hong S., Sycks D., Chan H.F., Lin S., Lopez G.P., Guilak F., Leong K.W., Zhao X. (2015). 3D printing of highly stretchable and tough hydrogels into complex, Cellularized structures. Adv. Mater..

[16] Fu Z., Angeline V., Sun W. (2021). Evaluation of printing parameters on 3D extrusion printing of pluronic hydrogels and machine learning guided parameter recommendation. Int. J. Bioprint..

[17] Tigner T.J., Rajput S., Gaharwar A.K., Alge D.L. (2019). Comparison of photo cross linkable gelatin derivatives and initiators for three-dimensional extrusion bioprinting. Biomacromolecules.

[18] Nichol J.W., Koshy S.T., Bae H., Hwang C.M., Yamanlar S., Khademhosseini A. (2010). Cell-laden microengineered gelatin methacrylate hydrogels. Biomaterials.

[19] Lessone S. (2017). Processi di Fotopolimerizzazione per L’ottenimento di un Nanocomposito a Base Cellulosica. Ph.D. Thesis.

[20] Chen Y.C., Lin R.Z., Qi H., Yang Y., Bae H., Melero-Martin J.M., Khademhosseini A. (2012). Functional human vascular network generated in photocrosslinkable gelatin methacrylate hydrogels. Adv. Funct. Mater..

[21] Pan J., Deng J., Yu L., Wang Y., Zhang W., Han X., Camargo P.H.C., Wang J., Liu Y. (2020). Investigating the repair of alveolar bone defects by gelatin methacrylate hydrogels-encapsulated human periodontal ligament stem cells. J. Mater. Sci. Mater. Med..

[22] Guimarães C.F., Gasperini L., Marques A.P., Reis R.L. (2020). The stiffness of living tissues and its implications for tissue engineering. Nat. Rev. Mater..

[23] Arani A., Arunachalam S.P., Chang I.C., Baffour F., Rossman P.J., Glaser K.J., Trzasko J.D., McGee K.P., Manduca A., Grogan M. (2017). Cardiac MR elastography for quantitative assessment of elevated myocardial stiffness in cardiac amyloidosis. J. Magn. Reson. Imaging.

[24] Bertassoni L.E., Cardoso J.C., Manoharan V., Cristino A.L., Bhise N.S., Araujo W.A., Zorlutuna P., Vrana N.E., Ghaemmaghami A.M., Dokmeci M.R. (2014). Direct-write bioprinting of cell-laden methacrylated gelatin hydrogels. Biofabrication.

[25] Celikkin N., Mastrogiacomo S., Jaroszewicz J., Walboomers X.F., Swieszkowski W. (2018). Gelatin methacrylate scaffold for bone tissue engineering: The influence of polymer concentration. J. Biomed. Mater. Res. A.

[26] Pepelanova I., Kruppa K., Scheper T., Lavrentieva A. (2018). Gelatin-methacryloyl (GelMA) hydrogels with defined degree of functionalization as a versatile toolkit for 3D cell culture and extrusion bioprinting. Bioengineering.

[27] Shie M.Y., Lee J.J., Ho C.C., Yen S.Y., Ng H.Y., Chen Y.W. (2020). Effects of gelatin methacrylate bio-ink concentration on mechano-physical properties and human dermal fibroblast behavior. Polymers.

[28] Yin J., Yan M., Wang Y., Fu J., Suo H. (2018). 3D bioprinting of low-concentration cell-laden gelatin methacrylate (GelMA) bioinks with a two-step cross-linking strategy. ACS Appl. Mater. Interfaces.

[29] Xu P., Guan J., Chen Y., Xiao H., Yang T., Sun H., Wu N., Zhang C., Mao Y. (2020). Stiffness of photocrosslinkable gelatin hydrogel influences nucleus pulposus cell propertiesin vitro. J. Cell. Mol. Med..

[30] Wei K., Sun J., Gao Q., Yang X., Ye Y., Ji J., Sun X. (2021). 3D “honeycomb” cell/carbon nanofiber/gelatin methacryloyl (GelMA) modified screen-printed electrode for electrochemical assessment of the combined toxicity of deoxynivalenol family mycotoxins. Bioelectrochemistry.

[31] Li J., Moeinzadeh S., Kim C., Pan C.C., Weale G., Kim S., Abrams G., James A.W., Choo H., Chan C. (2023). Development and systematic characterization of GelMA/alginate/PEGDMA/xanthan gum hydrogel bioink system for extrusion bioprinting. Biomaterials.

[32] Martyniak K., Lokshina A., Cruz M.A., Karimzadeh M., Kemp R., Kean T.J. (2022). Biomaterial composition and stiffness as decisive properties of 3D bioprinted constructs for type II collagen stimulation. Acta Biomater..

[33] Di Giuseppe M., Law N., Webb B., Macrae R.A., Liew L.J., Sercombe T.B., Dilley R.J., Doyle B.J. (2018). Mechanical behaviour of alginate-gelatin hydrogels for 3D bioprinting. J. Mech. Behav. Biomed. Mater..

[34] Ouyang L., Yao R., Zhao Y., Sun W. (2016). Effect of bioink properties on printability and cell viability for 3D bioplotting of embryonic stem cells. Biofabrication.

[35] Zhu W., Qu X., Zhu J., Ma X., Patel S., Liu J., Wang P., Lai C.S., Gou M., Xu Y. (2017). Direct 3D bioprinting of prevascularized tissue constructs with complex microarchitecture. Biomaterials.

[36] Semba J.A., Mieloch A.A., Tomaszewska E., Cywoniuk P., Rybka J.D. (2022). Formulation and evaluation of a bioink composed of alginate, gelatin, and nanocellulose for meniscal tissue engineering. Int. J. Bioprint..

[37] Bedada F.B., Chan S.S., Metzger S.K., Zhang L., Zhang J., Garry D.J., Kamp T.J., Kyba M., Metzger J.M. (2014). Acquisition of a quantitative, stoichiometrically conserved ratiometric marker of maturation status in stem cell-derived cardiac myocytes. Stem. Cell Rep..

[38] Pioner J.M., Vitale G., Steczina S., Langione M., Margara F., Santini L., Giardini F., Lazzeri E., Piroddi N., Scellini B. (2023). Slower calcium handling balances faster cross-bridge cycling in human MYBPC3 HCM. Circ. Res..

[39] Doss M.X., Di Diego J.M., Goodrow R.J., Wu Y., Cordeiro J.M., Nesterenko V.V., Barajas-Martínez H., Hu D., Urrutia J., Desai M. (2012). Maximum diastolic potential of human induced pluripotent stem cell-derived cardiomyocytes depends critically on IKr. PLoS ONE.

[40] Pioner J.M., Santini L., Palandri C., Martella D., Lupi F., Langione M., Querceto S., Grandinetti B., Balducci V., Benzoni P. (2019). Optical investigation of action potential and calcium handling maturation of hiPSC-cardiomyocytes on biomimetic substrates. Int. J. Mol. Sci..

[41] Coppini R., Ferrantini C., Yao L., Fan P., Del Lungo M., Stillitano F., Sartiani L., Tosi B., Suffredini S., Tesi C. (2012). Late sodium current inhibition reverses electromechanical dysfunction in human hypertrophic cardiomyopathy. Circulation.

[42] Goversen B., van der Heyden M.A., van Veen T.A., de Boer T.P. (2018). The immature electrophysiological phenotype of iPSC-CMs still hampers in vitro drug screening: Special focus on IK1. Pharmacol. Ther..

[43] Moreau A., Mercier A., Thériault O., Boutjdir M., Burger B., Keller D.I., Chahine M. (2017). Biophysical, molecular, and pharmacological characterization of voltage-dependent sodium channels from induced pluripotent stem cell-derived cardiomyocytes. Can. J. Cardiol..

